# Integrating Home-Based Palliative Care Into Primary Healthcare in Jodhpur, Rajasthan: Stakeholder-Perceived Barriers, Facilitators, and System Needs

**DOI:** 10.7759/cureus.112125

**Published:** 2026-07-06

**Authors:** Bhargaviba Jadeja, Srikanth Srinivasan, Manoj Kamal, Pankaj Bhardwaj, Manoj Kumar Gupta, Anjum Khan Jaod, Bharat Paliwal, Latha A

**Affiliations:** 1 Department of Community Medicine and Family Medicine, All India Institute of Medical Sciences (AIIMS), Jodhpur, Jodhpur, IND; 2 Department of Anaesthesiology and Critical Care, All India Institute of Medical Sciences (AIIMS), Jodhpur, Jodhpur, IND; 3 Department of Indian Council of Medical Research (ICMR), National Institute for Implementation Research on Non-Communicable Diseases (NIIRNCD), Jodhpur, IND; 4 Department of Anaesthesia, Bhagwan Mahaveer Cancer Hospital and Research Centre, Jaipur, IND; 5 College of Nursing, All India Institute of Medical Sciences (AIIMS), Jodhpur, Jodhpur, IND

**Keywords:** community health workers, health services integration, home care services, palliative care, primary health care

## Abstract

Background and objective

Home-based palliative care (HBPC) can reduce suffering and improve the quality of life for patients with serious chronic illnesses. However, its integration into routine government primary healthcare remains limited. Understanding health system readiness and provider perspectives is essential for its feasible integration. This study aimed to explore the barriers, facilitators, and stakeholder recommendations for integrating HBPC into urban Primary Healthcare Centers (PHCs) in Jodhpur, Rajasthan.

Methods

A qualitative study was conducted using purposive sampling. Data were collected through Key Informant Interviews (KIIs) with district health administrators and a palliative care physician, In-Depth Interviews (IDIs) with Medical Officers, and Focus Group Discussions (FGDs) with frontline workers, including Accredited Social Health Activists (ASHAs) and Auxiliary Nurse Midwives (ANMs). A total of 30 participants were included. Data were analyzed using thematic analysis.

Results

Barriers were identified at two levels. Knowledge and behavioral barriers included limited awareness and confidence among providers, low community awareness, sociocultural hesitation, and follow-up challenges. Structural barriers included a lack of formal PHC-level programs, weak referral pathways, limited availability of essential medicines such as morphine, high workload among frontline workers, and logistical constraints. Facilitators included community trust in ASHAs and ANMs, availability of basic medicines, referral linkages with tertiary care centers, and willingness for training and integration.

Conclusion

Integration is limited by gaps in provider capacity, medicine access, and service structure. Existing community trust and referral linkages provide a foundation for integration. Strengthening structured pathways, workforce capacity, and access to essential medicines is required for effective integration.

## Introduction

Palliative care is an approach that aims to improve the quality of life of patients and their families living with life-threatening illnesses by preventing and alleviating suffering through the early identification and effective management of pain and other physical, psychosocial, and spiritual concerns. The need for palliative care has been steadily increasing worldwide due to aging populations and the growing burden of non-communicable diseases. However, despite this rising demand, access to palliative care remains markedly inequitable, particularly in low- and middle-income countries, where services are limited and largely confined to tertiary hospitals [1].

India bears a high burden of severe health-related suffering and palliative care needs [1]. A systematic review conducted in India estimated that approximately 7-10 million people require palliative care each year, but fewer than 4% receive these services [2]. Furthermore, population-based analyses have demonstrated a substantial need for palliative care among individuals with both cancer and non-cancer conditions [3]. Studies evaluating community-based programs in India have shown that home-based and primary-level palliative care models can reduce unnecessary hospitalizations, improve symptom control, and provide more equitable and culturally acceptable care. Together, these findings underscore the need to strengthen community- and home-based palliative care (HBPC) models within the Indian health system [3,4].

To address the existing gaps in access, the Government of India launched the National Programme for Palliative Care (NPPC) under the National Health Mission to strengthen palliative care services at district-level hospitals and facilitate their integration into primary and community health care. The program emphasizes capacity building among health professionals, community awareness, and linking palliative care with existing health services to ensure continuity of care closer to patients’ homes, thereby improving symptom management and quality of life [5]. Similarly, national and international public health recommendations advocate integrating palliative care into primary care systems to enhance the quality of life of patients who may lack access to tertiary care facilities, particularly for effective symptom management [6].

However, the integration of palliative care across India remains inconsistent. Inadequate training and insufficient confidence among primary-level healthcare providers and frontline workers in identifying, counseling, and providing follow-up care for palliative patients in the community have been recognized as key barriers to access to high-quality palliative care services [7]. Furthermore, limited availability of essential controlled medications, such as morphine, at peripheral health facilities hinders regular home follow-up visits and compromises continuity of care for patients with severe illness or those who are homebound [8].

Rajasthan has recently launched state-supported home-based palliative care (HBPC) services for older adults and individuals with chronic illnesses, reflecting a positive policy commitment toward strengthening palliative care delivery in community settings [9]. The state has also implemented HBPC pilot programs at the district level, demonstrating the gradual expansion of service models beyond the hospital setting. Jodhpur district, which hosts an active tertiary palliative care unit and has initiated home-based care services, provides a valuable setting for exploring strategies to integrate home-based palliative care into government health systems [5]. However, evidence regarding the preparedness of District-Level Administrators (DLAs), Medical Officers, Auxiliary Nurse Midwives (ANMs), and Accredited Social Health Activists (ASHAs), as well as the practical barriers and facilitators to this integration, remains limited [10].

Research documenting stakeholder perspectives plays a crucial role in informing the design of effective integration pathways, because it is not sufficient to have a clinical protocol that is simply implemented - it is also essential to examine system capacity, medicine availability, workforce adequacy, referral linkages, and community acceptance of such integration. Therefore, the current study aimed to investigate the barriers, facilitators, and stakeholder recommendations for integrating HBPC within the existing urban government primary healthcare system in Jodhpur district, Rajasthan. This study sought to examine health system- and provider-level factors influencing the integration of HBPC into urban Primary Healthcare Centers (PHCs) in Jodhpur, Rajasthan. It further aimed to elicit stakeholder recommendations for strengthening implementation within the government primary healthcare system.

## Materials and methods

Study design

A qualitative implementation research design was adopted to identify system-level and provider-level determinants that influence the integration of HBPC into the government health system. This design enabled an in-depth exploration of experiences, perceptions, and challenges associated with service delivery. Qualitative methods were chosen to capture the contextual factors shaping program implementation. The approach facilitated the inclusion of diverse perspectives from stakeholders involved in planning, managing, and delivering care, and thus generated actionable insights regarding barriers, facilitators, and potential strategies for integrating HBPC within primary healthcare services.

Study setting

The study was conducted in Jodhpur district, Rajasthan, and encompassed urban primary health centres as well as the district health administrative system. This setting was selected because palliative care outpatient services were already being provided at a tertiary government hospital in the district. In addition, the district health system has an established network of frontline health workers, including ANMs and ASHAs, who routinely conduct home visits under various national health programmes. The presence of both tertiary care services and community-based health workers created an appropriate context to explore opportunities and challenges related to the integration of HBPC.

Study participants

Participants were drawn from key stakeholder groups across different levels of the government health system involved in planning and delivering healthcare services. These included district health administrators who were responsible for programme implementation, a tertiary care physician providing palliative services, Medical Officers working in urban primary health centres, and frontline health workers, including ANMs and ASHAs. Eligibility was based on current involvement in health service delivery or administration, as well as willingness to participate. The inclusion of participants from administrative, clinical, and frontline cadres enabled a comprehensive assessment of system readiness, operational barriers, and practical considerations influencing the integration of HBPC services.

Sampling technique

Purposive sampling was used to recruit participants directly involved in healthcare planning, administration, or service delivery relevant to the integration of palliative care. This method allowed the selection of individuals who had relevant expertise and experience within the health system. Representation was ensured across key stakeholder groups, including administrative authorities, Medical Officers, and frontline health workers, to capture diverse perspectives. A total of 303 stakeholders consented to participate and were included in the study. However, the exact number of individuals initially approached, as well as the rate of refusal or non-availability, were not formally documented during recruitment. By deliberately involving multiple levels of the health system, the study aimed to obtain balanced insights into both policy-level perspectives and frontline operational realities of HBPC services.

Data collection

Data were collected through multiple qualitative methods to obtain in-depth perspectives from diverse stakeholder groups. Key Informant Interviews (KIIs) with district administrators and the tertiary care physician were conducted to gather policy- and systems-level information. In-Depth Interviews (IDIs) were conducted with healthcare delivery staff, including Medical Officers, to explore facility preparedness, referral systems, medicine availability, and service delivery capacity. Focus Group Discussions (FGDs) were conducted separately with ANMs and ASHAs to capture field-level experiences, community-related challenges, training needs, and workload-related issues. All sessions were conducted with informed consent, audio-recorded, transcribed verbatim, anonymized, and supplemented with field notes.

The detailed interview guides used for KIIs, IDIs, and FGDs, including their probes, are provided in the Appendices. The KII, IDI, and FGD guides were developed to address key domains relevant to HBPC integration, including awareness, service availability, referral pathways, workforce training, medicine access, logistical barriers, community acceptance, digital support, and stakeholder recommendations. The guides were reviewed for alignment with the study objectives before data collection; however, they were not formally pilot-tested before implementation. This has been acknowledged as a methodological limitation.

Data analysis

Thematic analysis was employed to analyze data obtained from KIIs, IDIs, and FGDs. Transcripts and field notes were reviewed concurrently with data collection to assess data saturation and to facilitate preliminary coding. Saturation was considered achieved when additional interviews and discussions within each stakeholder group did not generate new or substantively different codes, categories, or themes relevant to the study objectives, and when the identified barriers, facilitators, and system needs became consistently observed across administrative, clinical, and frontline worker groups. The analysis was conducted manually, without the use of qualitative data analysis software such as NVivo or Atlas.ti. Transcripts were read repeatedly to ensure familiarization, followed by open coding to identify meaningful text segments related to barriers, facilitators, training needs, digital support, and system-level recommendations.

Similar codes were grouped into broader categories and then organized into major and minor themes. Coding and theme development were reviewed within the investigator team, and differences in interpretation were resolved through consensus during theme refinement. Thematic categories were checked against transcripts, field notes, and stakeholder groups to improve internal consistency and ensure that the final themes reflected administrative, clinical, and frontline worker perspectives. Formal respondent validation or member checking was not performed after theme development, and this has been acknowledged as a limitation.

Ethical considerations

Ethical approval for the present study was obtained from the Institutional Ethics Committee (IEC) of the All India Institute of Medical Sciences (AIIMS), Jodhpur. The research project was approved under Certificate Reference Number AIIMS/IEC/2023/4867, following review and discussion during the IEC meeting held on December 14, 2023. Participation in the study was voluntary, and written informed consent was obtained from all participants before interviews and focus group discussions. Confidentiality was maintained by removing personal identifiers from transcripts, and all data were securely stored and managed in accordance with institutional ethical guidelines.

## Results

Participant profile

A total of 303 stakeholders from the government health system were included in the study through KIIs, IDIs, and FGDs. The participants spanned multiple tiers of the health system, including district health administrators, tertiary care physicians, Medical Officers from urban primary health centers, and frontline health workers (ANMs and ASHAs). This range of participants enabled the study to capture perspectives across administrative, clinical, and community-level service delivery domains. The breakdown of participants across stakeholder groups and data collection methods is presented in Table 1.

**Table 1:** Participant distribution by stakeholder and method KII: Key Informant Interview; IDI: In-Depth Interview; FGD: Focus Group Discussion; UPHC: Urban Primary Health Center; ANM: Auxiliary Nurse Midwife; ASHA: Accredited Social Health Activist

Stakeholder group	Number	Method
Health administrators	2	KII
Tertiary care physician	1	KII
Medical Officers (UPHCs)	16	IDI
ANMs	135	21 FGDs
ASHAs	149	21 FGDs

Common/knowledge-behavioral barriers

Stakeholders explained several barriers that impact the integration of HBPC in the government's primary healthcare system. These barriers occurred at two general levels: common or knowledge-behavioral barriers and structural or health-system barriers. Participants from various cadres identified gaps in awareness, training, and community understanding as well as operational constraints within the health system that affected the feasibility of regular HBPC delivery.

Limited Awareness and Confidence Among Providers

There were several ASHAs, ANMs, and Medical Officers who reported that their knowledge of palliative care was limited before recent training sessions. Many participants noted that they had previously referred such patients to higher-level facilities without providing symptom management or counseling at the primary care level. One participant stated, “We learned about palliative care for the first time during this training” (ASHA/ANM, FGD). Tertiary care physicians also reported limited preparedness and confidence among peripheral health staff in managing symptoms of patients requiring palliative care at the community level.

Poor Awareness of NPPC

Limited awareness of NPPC was also reported by a number of frontline workers, as well as some Medical Officers. Participants indicated that program implementation mechanisms at the PHC level were not clear, which resulted in unclear roles and service pathways. One of the Medical Officers commented, “A dedicated system should be established specifically for the effective implementation of palliative care policy.” Another Medical Officer stated, “I do not have much awareness about the existing policies.”

Low Public Awareness and Sociocultural Hesitation

Frontline workers often explained issues of low community knowledge around palliative care. Families were often alien to the concept of supportive care for chronic or terminal illnesses and even resisted counseling or follow-up. Cultural perceptions about illness, caregiving responsibilities, and delayed health-seeking behavior were also reported as factors impacting continuity of care. Participants described problems faced in home visits and counseling interactions. One participant explained, “It is difficult to encourage families to spend time with the patient, as many are unable or unwilling due to their responsibilities and time constraints" (ASHA, ANM). Another participant reported, “The biggest difficulty was that the patient’s family members did not support me at all” (ASHA, ANM).

Participants also mentioned examples of families who were not willing to accept the need for supportive care. One respondent noted, “Sometimes patients and their families do not accept that any kind of care is needed” (ASHA, ANM). Similarly, another participant stated, “It is challenging to explain to families that such care exists and to prepare them to accept it” (ASHA, ANM). Resistance from patients themselves was also described. A frontline worker explained, “Sometimes when we visit, patients become irritated and question who informed us about their illness, and ask us to leave” (ASHA, ANM). Another participant added, “Some people do not even open the door” (ASHA, ANM).

Structural/health-system barriers

Participants also characterized several structural and health-system level barriers that limit the integration of HBPC into primary healthcare settings. These barriers were related to the organization of services, referral systems, availability of medicines, workload of the workforce, and logistical support.

Lack of a Formal PHC-Level Home-Based Palliative Care Service Delivery

Administrators and Medical Officers said that structured HBPC services are not yet formally part of routine PHC/sub-center activities. As a consequence, palliative care at the primary care level is confined mostly to identification and referral as opposed to structured service delivery. One Medical Officer noted, “Home-based palliative care services have not yet been initiated."

Weak Referral and Reporting Pathways

Medical Officers mentioned the lack of a formal mechanism of referral and reporting of palliative care cases at the PHC level. In most cases, referrals are informal and involve a written slip to take patients to a higher-level facility. One Medical Officer explained, “Only routine painkillers are prescribed here; dedicated palliative care medicines are not available at this level and are provided only at tertiary care centers.” Another Medical Officer added, “When a palliative care patient presents, we try to provide counseling; however, if the case is more severe, we are unable to prescribe morphine or similar medications, and such patients need to be referred to higher-level facilities."

Limited Availability of Essential Medicines, Especially Morphine

Medical Officers emphasized the lack of availability of essential palliative medicines, especially morphine and other strong opioids, at peripheral facilities due to regulatory and supply issues. As a result, patients with severe pain have to be referred to the tertiary centers for treatment. One Medical Officer stated, “Only routine painkillers are prescribed here; dedicated palliative care medicines are not available at this level and are provided only at tertiary care centers”.

Insufficient System Capacity and Funding

Participants also noted the limitations of infrastructure, workforce capacity, and financial resources at peripheral facilities. These constraints hinder the capacity of the health system to organize regular home visits or offer comprehensive palliative services at the community level. One of the Medical Officers put it this way, “We do not have sufficient facilities to provide adequate treatment.”

Heavy Workload of ASHAs and ANMs

Both Medical Officers and on-the-ground workers have reported that ASHAs and ANMs are already burdened with multiple national health program activities, such as in maternal health services, NCD-related programs, community surveys, and extensive digital reporting duties. This workload means less time is left for palliative care visits and counseling. One Medical Officer noted, “ANMs and ASHA workers already have a substantial workload, including identifying pregnant women, managing non-communicable disease-related tasks, conducting surveys for seasonal illnesses, and fulfilling other government-mandated responsibilities.”

Frontline workers also described time constraints during service delivery. Participants stated, “We are unable to spend sufficient time with patients for detailed discussions” (ASHA/ANM). “We do not have enough time ourselves” (ASHA/ANM); and “now everything is online; we spend long hours completing entries and reports, sometimes until late at night" (ASHA/ANM).

Transport and Logistics Difficulty for Home Visits

Participants also raised issues of transport and logistical barriers to home-based service delivery. Frontline workers said it is challenging to reach households and ensure the medicines are delivered without dedicated travel support. One participant mentioned, “We are not able to deliver medicines to patients ourselves” (ASHA/ANM, FGD).

Lack of Incentives or Payment for Home-Based Work

Another barrier reported by both frontline workers and administrators was a lack of specified incentives or financial support in terms of conducting palliative home visits. Participants felt that without incentives or recognition mechanisms, it is not easy to sustain follow-up and regular home visits within the present structure of workload.

Training and mentorship requirements

Stakeholders strongly emphasized the need for uninterrupted training and supportive supervision of PHC-level teams for palliative care delivery. Participants indicated that many frontline providers currently have insufficient training and practical exposure on how to deal with palliative care cases at the community level. Medical Officers emphasized that specialized training and continued mentoring of other health professionals by experienced palliative care professionals are vital in developing confidence in symptom control, counseling, and follow-up care. Participants also noted that there is still incomplete coverage of existing training across the district. One participant explained, “Community Health Officers (CHOs) received training during induction, and ANMs and ASHAs have received partial training; however, this has not yet been fully implemented across the entire district.” Another Medical Officer stated, “I have not received any formal training in palliative care; I was only exposed to it briefly during my undergraduate studies.”

Facilitators supporting integration

Despite the barriers identified, stakeholders also described several existing facilitators that could support the integration of HBPC within the government health system. Participants mentioned that certain aspects of palliative care are already being practised in an indirect manner in the form of routine home visits, the management of basic symptoms, family counseling, and referral of severe cases to referral centers at higher centers. The availability of basic medicines for relief of symptoms at PHC, functional referral linkages with AIIMS and other tertiary hospitals, and the community trust in ASHA and ANM home visits were identified as important enabling factors. Participants also mentioned better coordination between AIIMS and the Medical and Health Department as supportive of integration. One participant noted, “ASHA and ANM workers are already well accepted within the community and can identify seriously ill patients.” A Medical Officer explained, “If the case is more serious, we refer the patient to AIIMS or another higher-level hospital.”

Need for IT/digital support

Several stakeholders showed interest in using digital health tools to reinforce the HBPC services. Telemedicine platforms and mobile-based follow-up systems were considered as potential solutions for improving consultation, monitoring of symptoms and continuity of care for home-based patients. Participants recommended that the use of digital tools might support frontline workers to connect with experts when helping to manage complex cases, especially in remote or hard-to-reach areas. Such systems were also perceived as useful to enhance communication between PHCs and tertiary care centers. One participant explained, “Telemedicine and platforms such as Tele-MANAS are already operational. For seriously ill patients, consultations can be facilitated by linking with district hospitals.”

Stakeholder suggestions

Stakeholders offered several practical recommendations to strengthen the integration of HBPC into the primary healthcare system. These included increasing public awareness about palliative care, improving the implementation of existing policies, and developing standardized protocols for home visits and patient follow-up. Participants also emphasized the importance of ensuring the availability of essential medicines, including morphine, at peripheral facilities. Additional recommendations included providing mobility support, introducing team-based incentives for frontline workers, and establishing a dedicated nodal team to coordinate, monitor, and mentor palliative care activities at the district level. One participant suggested, “ASHA and ANM workers should be provided with appropriate incentives; however, the entire responsibility should not be placed solely on them. A dedicated nodal team is required to ensure effective coordination.” Another Medical Officer noted, “If both transport and medication support are ensured, home visits can be conducted more regularly.”

## Discussion

This study examined the stakeholder views on the inclusion of HBPC into PHC systems - specifically barriers, training requirements, facilitators, digital support, and operational recommendations. The findings raise some behavioral and system-level issues that affect the implementation of community-based palliative care through government health services. These results are consistent with findings from India and elsewhere internationally, which report on similar structural and operational barriers to scaling up HBPC services.

Knowledge-related barriers emerged as a major challenge among frontline providers. Many ASHAs, ANMs, and Medical Officers reported limited knowledge of and training in palliative care, with some participants indicating that they had first encountered the concept during recent training activities. This limited exposure reduced their confidence in symptom management and the provision of home-based care, resulting in frequent referrals to tertiary hospitals. Similar findings have been reported internationally, where inadequate training and the absence of well-defined referral systems have undermined primary care providers’ confidence in delivering palliative care [11,12]. Additionally, fragmented referral pathways and poor communication between hospital and community services have been recognized globally as major barriers to effective palliative care delivery [13,14]. In the Indian context, palliative care services outside Kerala remain largely hospital-centered, while low community awareness, sociocultural hesitation to discuss terminal illness, and inconsistent policy implementation further hinder continuity of care [15,16].

Structural barriers within the health system were identified as additional significant challenges. Participants reported that formal HBPC programs have not yet been institutionalized at the PHC and sub-center levels. Referral pathways remain informal, reporting systems for palliative care cases are largely absent, and dedicated funding for home visits remains limited. Similar gaps have been reported in other Indian studies, where inadequate coordination, unclear role definitions, and weak documentation systems have hindered continuity of HBPC services [17]. Limited availability of essential medicines, particularly morphine and other opioids, emerged as another key barrier. Although national policies aim to improve access to opioids, their implementation at peripheral facilities remains inconsistent [11,18]. Stakeholders also highlighted high workloads among ASHAs and ANMs, workforce shortages, the absence of incentives for home visits, and inadequate transport and logistical support. These findings are consistent with recommendations from the Indian Association of Preventive and Social Medicine, which highlight the need for incentives, supervision, and sustainable financing to support community-based palliative care programs [19].

The study also identified a substantial need for ongoing training and mentorship for PHC teams. Stakeholders emphasized the importance of structured training, refresher courses, and mentorship from palliative care specialists to strengthen confidence in symptom assessment, counseling, and home-based follow-up. Previous studies have demonstrated that targeted capacity-building programs effectively enhance palliative care delivery by primary healthcare providers [11,20]. Similarly, international evidence indicates that mentorship and specialist support models improve care coordination and promote person-centered care, particularly in resource-limited settings [19,21]. These findings suggest that sustained mentorship, rather than one-time training, is essential for strengthening palliative care services at the primary healthcare level.

Despite these barriers, a number of facilitators for integration were identified. Routine home visits by ASHAs and ANMs, availability of basic medicines for symptom relief at PHCs, connectivity of referrals with tertiary centers such as AIIMS, and high levels of community trust in frontline health workers are a basis for community-based palliative care. Evidence from Kerala has shown that community-linked home-care programs have a significant impact on improving access and quality of life in patients who require palliative care [22,23]. The findings from Jodhpur both align with and differ from Kerala’s community-linked palliative care experience. Similar to Kerala-based models, this study identified community trust in frontline workers, routine home visits, family counseling, and referral linkages as important facilitators for home-based care.

However, unlike the more established Kerala models, stakeholders in Jodhpur reported that PHC-level HBPC remains less formalized, with weaker reporting systems, limited opioid availability at peripheral facilities, no dedicated home-care workflow, and insufficient incentives or transport support for frontline workers. These differences suggest that replication of Kerala-style community palliative care models in Rajasthan would require adaptation to local health-system capacity, workforce workload, financing mechanisms, and medicine-access constraints. Similar experiences from other low-resource settings also demonstrate the effectiveness of community health workers to provide HBPC services using task-sharing approaches [24,25].

Stakeholders also emphasized the potential role of digital technologies in strengthening service delivery. Telemedicine platforms and mobile-based follow-up systems were seen as practical implementations to monitor symptoms, maintain communication with specialists, and enhance continuity of care in home-based patients [26]. Studies in India have demonstrated that the role of tele-mentoring and digital learning platforms in healthcare has been an important development in improving palliative care training and service delivery [27,28]. Integration with national digital health initiatives may also further promote patient monitoring and specialist consultation in real-time [29,30].

**Figure 1:**
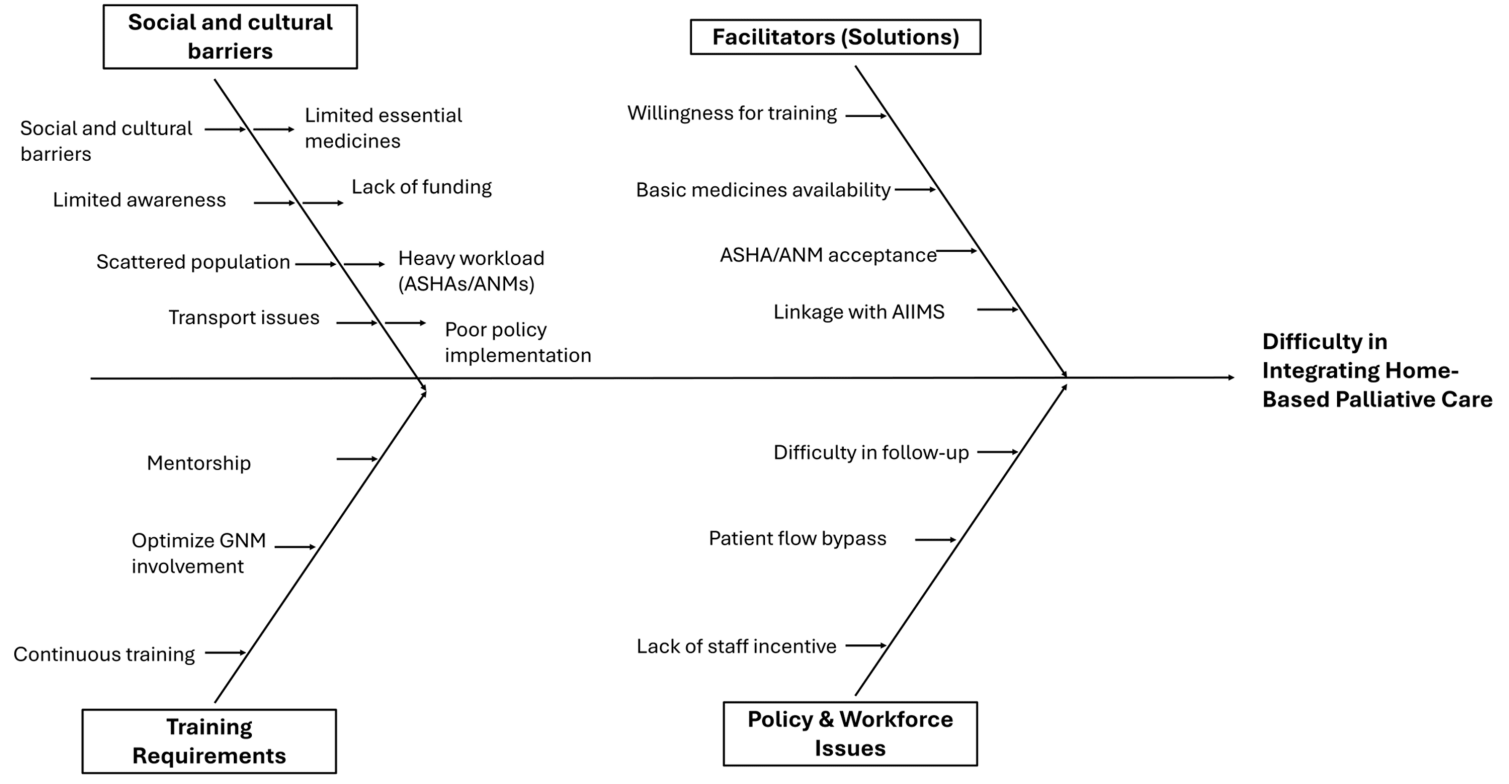
Stakeholder-perceived barriers, facilitators, and system needs ASHA: Accredited Social Health Activist; ANM: Auxiliary Nurse Midwife Created by authors using Microsoft PowerPoint

Participants also suggested several practical measures to strengthen integration, such as raising community awareness, as well as improving policy implementation at the local level, ensuring access to essential medicines, mobility support for visits at home and access to incentives for frontline workers [31,32]. The setting up of a highly dedicated nodal coordination unit for monitoring and mentorship was also suggested. Similar recommendations have been reported in the literature and include policy support, caregiver involvement, operational frameworks and workforce incentives as key components of effective HBPC systems [33,34]. These findings suggest the need to strengthen training, resources, coordination and digital support mechanisms to provide sustainable community-based palliative care services in the Indian primary healthcare system. An overview of our qualitative study is illustrated in Figure 1.

Limitations and future directions

This study is based on views from stakeholders from one district, and the majority of the participants represented urban health system settings. Therefore, the results should be interpreted with caution when considering their applicability to other geographic or health system settings. The study focused mainly on healthcare providers and administrators; however, the opinions of patients and caregivers did not fall within the scope of the research. Additionally, the findings were based on self-reported experiences and perceptions of participants, which might not reflect all operational challenges that arise during real-world delivery of HBPC services. Social desirability bias may also have influenced participant responses, particularly among frontline workers. ASHAs and ANMs may have underreported workload concerns, implementation challenges, or service delivery gaps because of perceived administrative expectations or concern about possible repercussions.

The KII, IDI, and FGD guides were developed based on the study objectives and relevant domains of HBPC integration; however, they were not formally pilot-tested before data collection. This may have limited the opportunity to refine question wording, sequencing, and probing before implementation. Thematic analysis was conducted manually without qualitative data analysis software. A formal independent multiple-coder procedure, intercoder reliability assessment, and respondent validation or member checking were not documented. These factors may have limited intercoder verification and participant confirmation of the final thematic interpretation.

Future research should include the perspectives of patients and caregivers to obtain a more comprehensive understanding of needs and experiences associated with HBPC. Multi-district or multi-state studies could provide further information on the feasibility of integrating palliative care into different health system settings. Implementation research related to pilot programs, digital health solutions, workforce training models, and access mechanisms related to opioids may help identify scalable programs for strengthening community-based palliative care within the primary healthcare system. Future studies should also consider pilot-testing qualitative interview guides, using formal multi-coder procedures, documenting intercoder agreement, and incorporating respondent validation to strengthen methodological rigor in qualitative implementation research.

## Conclusions

This qualitative implementation study suggests that integration of HBPC with the government health system of Jodhpur may be operationally achievable, based on stakeholder-perceived readiness, existing community-level workforce linkages, and referral connections with tertiary care; however, pilot implementation data are required before confirming feasibility. Major barriers include inadequate awareness among healthcare providers and the community, lack of a structured PHC-level HBPC framework, lack of availability of essential opioids such as morphine at peripheral facilities, heavy workload of ASHAs and ANMs, and transport and follow-up support gaps. These challenges impact the continuity and viability of community-based palliative care delivery. At the same time, there are several enabling factors that will provide a firm basis for integration within the existing health system. Community trust in ASHA and ANM home visits, availability of basic medicines for the relief of symptoms at PHCs, functional referral linkages with AIIMS and other centers at the tertiary levels, and improved coordination between different levels of the health system support the potential for program expansion. Strengthening integration would mean, among others, the establishment of a clear HBPC workflow under the NPPC, continuous training and mentorship of PHC teams, better access to necessary medicine and opioids, digital/telemedicine-based follow-up systems, and mobility support and team-based incentives for frontline workers.

## Appendices

The detailed IDI guide used for Medical Officers is presented in Table 2.

**Table 2:** IDI guide - Medical Officers IDI: In-Depth Interview; NPPC: National Programme for Palliative Care; OPD: outpatient department

S. no	Question	Probes
1	Have you heard about palliative care? What is your understanding of it?	Concept clarity, scope
2	What medical conditions require palliative care?	Cancer, chronic illness, geriatric
3	When should palliative care be initiated?	Early vs. late stage
4	Are there palliative care patients in your facility?	Case identification
5	Describe treatment and referral practices	Referral pathways
6	Have you undergone training in palliative care?	Type, duration
7	Is your staff trained?	Skill gaps
8	What challenges do you foresee?	Resources, system issues
9	Do you feel confident managing such patients?	Clinical confidence
10	Is oral morphine available? Any concerns?	Opioid access, apprehension
11	Is there a palliative care OPD?	Service availability
12	Are you aware of policies/laws?	NPPC awareness
13	Recommendations for implementing NPPC	Practical suggestions

The FGD guide used for ASHAs and ANMs is presented in Appendix Table 3.

**Table 3:** FGD guide - ASHAs and ANMs FGD: Focus Group Discussion; ASHA: Accredited Social Health Activist; ANM: Auxiliary Nurse Midwife

S. no	Question	Probes
1	Do you know about palliative care?	Awareness
2	Have you provided home care to terminally ill patients?	Experience
3	What difficulties did you face?	Logistics, family cooperation
4	What are your views on caregiver attitudes?	Support, resistance
5	Have you received training?	Training gaps

The KII guide used for district-level stakeholders is presented in Table 4.

**Table 4:** KII guide - district stakeholders KII: Key Informant Interview; NPPC: National Programme for Palliative Care; ASHA: Accredited Social Health Activist; ANM: Auxiliary Nurse Midwife; MO: Medical Officer; NGO: non-governmental organization; PPP: public-private partnership

Domain	Key questions	Probes
Status of services	Current status of palliative care in the district	Availability, coverage
Importance	Importance of home-based care	Public health relevance
System functioning	Identification, treatment, follow-up	Workflow
Policy environment	Policies/guidelines availability	NPPC implementation
Workforce	Training status of ASHA/ANM/MO	Capacity gaps
Medicines	Availability of opioids	Supply barriers
Referral systems	Effectiveness of referral pathways	Coordination gaps
Infrastructure	Transport, home visits, telemedicine	Resource needs
Sociocultural factors	Community awareness, stigma	Acceptance
Challenges	Administrative and operational barriers	Funding, logistics
Solutions	Policy and system improvements	Integration strategies
Technology	Role of telemedicine	Follow-up support
Partnerships	NGOs, the private sector role	PPP models
Recommendations	Key suggestions for integration	Policy + implementation
